# Supplementary material for: Precise temporal control of neuroblast migration through combined regulation and feedback of a Wnt receptor
Source: eLife. 2023 May 15;12:e82675. doi: 10.7554/eLife.82675 (PMC10259474; doi:10.7554/eLife.82675)
Supplement: Supplementary file 1. — a. CRISPR/Cas9-mediated gene edits of mig-1 and cdk-1. b. C. elegans strains used in this study. c. Single guide RNA (sgRNA) and single-stranded oligodeoxynucleotide (ssODN) sequences used for CRISPR/Cas9-mediated gene editing. [file elife-82675-supp1.docx]

**Supplementary file 1a**

**Details on CRISPR/Cas9-mediated gene edits of *mig-1* and *cdk-1***

| **CRISPR/Cas-9 allele** | **Description** |
| --- | --- |
| *mig-1*(*hu295* [Δintron A]) | CRISPR/Cas9 generated deletion in *mig-1* intron: 1846-1936 bp from the ATG |
| *mig-1*(*hu299* [Δintron B]) | CRISPR/Cas9 generated deletion in *mig-1* intron: 2773-2849 bp from the ATG |
| *mig-1*(*hu314* [Δupstream 1]) | CRISPR/Cas9 generated deletion in the *mig-1* upstream region: 2781-2572 bp from the ATG |
| *mig-1*(*hu315* [Δupstream 2]) | CRISPR/Cas9 generated deletion in the *mig-1* upstream region: 267-138 bp from the ATG |
| *mig-1*(*hu335* [Δupstream 1-2]) | CRISPR/Cas9 generated deletions in the *mig-1* upstream region: 2781-2572 bp and 267-138 bp from the ATG |
| *cdk-1*(*hu277* [*AID::cdk-1*]) | CRISPR/Cas9 generated 135 bp insertion at start of *cdk-1* ORF, replacing the ATG |

**Supplementary file 1b**

***C. elegans* strains used in this study**

| **Strains** | **Source** | **Identifier** |
| --- | --- | --- |
| N2 Bristol | *Caenorhabditis* Genetics Center | N2 |
| *heIs63[Pwrt-2::GFP::PH, Pwrt-2::GFP::H2B, Plin-48::mCherry]* | Wildwater et al., 2011 | SV1009 |
| *huIs166[Pwrt-2::mCherry::PH; Pwrt-2::mCherry::H2B; dpy-20(+]* | Rella et al., 2021 | KN2598 |
| *mig-1(hu295); huIs166[Pwrt-2::mCherry::PH; Pwrt-2::mCherry::H2B; dpy-20(+]* | This study | KN3071 |
| *mig-1(hu299); huIs166 [Pwrt-2::mCherry::PH; Pwrt-2::mCherry::H2B; dpy-20(+)]* | This study | KN3078 |
| *mig-1(hu314); heIs63[Pwrt-2::GFP::PH, Pwrt-2::GFP::H2B, Plin-48::mCherry]* | This study | KN3133 |
| *mig-1(hu315); heIs63[Pwrt-2::GFP::PH, Pwrt-2::GFP::H2B, Plin-48::mCherry]* | This study | KN3134 |
| *mig-1(hu335); heIs63[Pwrt-2::GFP::PH, Pwrt-2::GFP::H2B, Plin-48::mCherry]* | This study | KN3222 |
| *cdk-1(hu277[AID::cdk-1]); huIs210[Pegl-17::tir1::TagBFP, Pmyo-2::TdTomato]; heIs63[Pwrt-2::GFP::PH, Pwrt-2::GFP::H2B, Plin-48::mCherry]* | This study | KN2936 |
| *ced-3(n717); heIs63[Pwrt-2::GFP::PH, Pwrt-2::GFP::H2B, Plin-48::mCherry]* | This study | KN2885 |
| *pig-1(gm344); heIs63[Pwrt-2::GFP::PH, Pwrt-2::GFP::H2B, Plin-48::mCherry]* | This study | KN2897 |
| *bar-1(ga80); heIs63[Pwrt-2::GFP::PH, Pwrt-2::GFP::H2B, Plin-48::mCherry]* | Mentink et al., 2014 | KN1739 |
| *huIs179[Pegl-17::ΔN-bar-1, Pmyo-2::mCherry]; heIs63[Pwrt-2::GFP::PH, Pwrt-2::GFP::H2B, Plin-48::mCherry]; mab-5(gk670)* | Rella et al., 2021 | KN2651 |

**Supplementary file 1c**

**sgRNA and ssODN sequences used for CRISPR/Cas9-mediated gene editing**

| **Name** | **Alleles** | **Sequence** |
| --- | --- | --- |
| sgRNA *dpy-10* | *cdk-1(hu277)* | GCUACCAUAGGCACCACGAG |
| sgRNA *pha-1* | *mig-1(hu314), mig-1(hu315), mig-1(hu335)* | AUGAAUAACUUGAUGAACAU |
| sgRNA *AID::cdk-1* | *cdk-1(hu277)* | AUAGGAUCCAUAACUAAAAU |
| sgRNA *mig-1* Δintron A forward primer | *mig-1(hu295)* | UUUAUUACGCAUCUCAUAUG |
| sgRNA *mig-1* Δintron A reverse primer | *mig-1(hu295)* | CAUACGGUCGCGAGGCCAUG |
| sgRNA *mig-1* Δintron B forward primer | *mig-1(hu299)* | UAGGCAGAGUGCACGGCGGG |
| sgRNA *mig-1* Δintron B reverse primer | *mig-1(hu299)* | AUAUCACUUGUCACGUCCUC |
| sgRNA *mig-1* Δupstream 1 forward primer | *mig-1(hu314), mig-1(hu335)* | UGUGAUGUAUCGUGGCAGAU |
| sgRNA *mig-1* Δupstream 1 reverse primer | *mig-1(hu314), mig-1(hu335)* | GGGGGGGGAACAACCACCGU |
| sgRNA *mig-1* Δupstream 2 forward primer | *mig-1(hu315), mig-1(hu335)* | AAGAGAGCUGAAAGAUGGAG |
| sgRNA *mig-1* Δupstream 2 reverse primer | *mig-1(hu315), mig-1(hu335)* | UGGGUCAUUUGUCAACGGAC |
| ssODN *dpy-10* | *cdk-1(hu277)* | CACTTGAACTTCAATACGGCAAGATGAGAATGACTGGAAACCGTACCGCATGCGGTGCCTATGGTAGCGGAGCTTCACATGGCTTCAGACCAACAGCCTAT |
| ssODN *pha-1* | *mig-1(hu314), mig-1(hu315), mig-1(hu335)* | TCTTAAAACAAACCATGAAGATTATGGTAATCAAAATACGAATCGAAGACTCAAAAAGAGTATGCTGTATGATTACAGATGTTCATCAAGTTATTCATAAATCATTGATAGGTTCAGATTGTAAGTCTTGATTATCTATC |
| ssODN *AID::cdk-1* | *cdk-1(hu277)* | TTGTTCTGACAAAATTCATTTATAATTTTAGTTATGCCTAAAGATCCAGCCAAACCTCCGGCCAAGGCACAAGTTGTGGGATGGCCACCGGTGAGATCATACCGGAAGAACGTGATGGTTTCCTGCCAAAAATCAAGCGGTGGCCCGGAGGCGGCGGCGTTCGTGAAGATGGATCCTATTCGCGAAGGAGAAGTGGCCCA |
| ssODN *mig-1* Δintron A | *mig-1(hu295)* | TAAGGTCTTGTAGGGAATTTTATTACGCATCTCATATGTAGCGCGCTTTTTCACTTTTTCCCTCTCAACT |
| ssODN *mig-1* Δintron B | *mig-1(hu299)* | CGGCAGTTGGACGGGAACCTGCGTCTCCACATCCCCTCATGCTCTCCGAGCACCACAAAACGCCCCTTTC |
| ssODN *mig-1* Δupstream 1 | *mig-1(hu314), mig-1(hu335)* | AGTTTTTGGAACTGATCCGTTGTCAGCCAGTCATCCGTCAGTGTCAGAGTCAACAAATCAGGGAAAACAC |
| ssODN *mig-1* Δupstream 2 | *mig-1(hu315), mig-1(hu335)* | TCCACCCCCCCCCCTCTCTTCCTCTTTCGCTTCTCGACAAGCCGTGGTGGTGCTGCCACTGGTGTGCCCC |

sgRNA, single guide RNA; ssODN, single-stranded oligodeoxynucleotide.
